# Childhood Vaccine Refusal: Sociodemographic, Behavioral, and Vaccine Confidence Factors in Konya, Türkiye

**DOI:** 10.3390/vaccines14060538

**Published:** 2026-06-17

**Authors:** Önder Aydemir, Elif Nur Yıldırım-Öztürk, Mehmet Koç

**Affiliations:** 1 Konya Provincial Health Directorate, Konya 42100, Türkiye; 2 Department of Radiation Oncology, Faculty of Medicine, Necmettin Erbakan University, Konya 42090, Türkiye

**Keywords:** vaccine refusal, vaccine hesitancy, trust in vaccines, childhood vaccination, case–control study, behavioral factors, COVID-19 vaccination, Türkiye

## Abstract

**Background/Objectives:** Childhood vaccine refusal may negatively affect immunization programs in Türkiye, where regional clusters of hesitancy have emerged despite high national coverage. This study aimed to identify sociodemographic, behavioral, and vaccine confidence-related factors independently associated with childhood vaccine refusal in Konya, Türkiye. **Methods:** An unmatched case–control study was conducted between July and October 2025 in family health centers across Konya. Cases were parents who had refused at least one routine childhood vaccine (*n* = 406); controls were parents whose children had completed all routine vaccinations (*n* = 412). Data were collected through face-to-face interviews using a 47-item structured questionnaire and the Turkish version of the Vaccine Hesitancy Scale (VHS). Independent associations were assessed using multivariable logistic regression, with multicollinearity evaluated by variance inflation factors. **Results:** Maternal employment (aOR = 0.371, 95% CI: 0.218–0.633), parental COVID-19 vaccination (aOR = 0.131, 95% CI: 0.086–0.200), mother’s complete childhood immunization (aOR = 0.418, 95% CI: 0.262–0.667), tetanus vaccination during pregnancy (aOR = 0.259, 95% CI: 0.159–0.421), and neonatal vitamin K administration (aOR = 0.256, 95% CI: 0.132–0.497) were independently associated with lower refusal odds. Higher number of children (aOR = 1.281) and perceived vaccine-related adverse events in the social environment (aOR = 16.982, 95% CI: 9.914–29.089) increased refusal odds. VHS scores were significantly lower in the refusal group (22.2 ± 6.4 vs. 39.8 ± 6.5; *p* < 0.001), indicating greater hesitancy. Notably, 21.9% of refusing parents reported being advised by a healthcare professional not to vaccinate. **Conclusions:** Childhood vaccine refusal in Konya was associated with sociodemographic, behavioral, preventive health-related, and vaccine confidence-related factors. The findings suggest relatively reduced engagement with selected preventive health practices, greater reliance on non-professional information sources, and lower vaccine confidence among refusing parents. Interventions should focus on strengthening healthcare-professional communication, trust-building, transparent risk communication, and evidence-based social media strategies.

## 1. Introduction

Vaccines are recognized as one of the most cost-effective and impactful public health interventions on a global scale, preventing millions of deaths each year [1]. Despite this, vaccine hesitancy and refusal have become serious issues that negatively affect immunization programs worldwide. The World Health Organization (WHO) identified vaccine hesitancy as one of the top ten threats to global health in 2019, and this issue has become even more pronounced with the COVID-19 pandemic [2].

Vaccine hesitancy is defined as a continuum ranging from full acceptance to complete refusal despite the availability of vaccines and is shaped by three main determinants: confidence, complacency, and convenience [3]. Vaccine hesitancy and refusal regarding childhood immunizations are particularly risky, as they largely depend on parental decisions. In many countries, the decline in vaccination rates has led to the re-emergence of preventable diseases such as measles [4].

Although national vaccination rates in Türkiye are generally high, local clusters of refusal and hesitancy observed in some regions may negatively impact both individual and community immunity [5,6].

Studies in Türkiye have reported the prevalence of vaccine hesitancy among parents ranging from 4.8% to 19.7%, with common reasons including safety concerns, distrust of vaccine manufacturers, and parental reliance on unreliable vaccine information sources [6,7].

Vaccine refusal may also be related to reduced acceptance of other preventive healthcare practices, including maternal vaccination during pregnancy, neonatal preventive interventions such as vitamin K administration and heel-prick screening, and COVID-19 vaccination [8,9]. Especially during the pandemic, distrust toward COVID-19 vaccines also negatively affected attitudes toward routine childhood vaccinations [9].

Konya is the largest province of Türkiye in terms of area, and with its urban and rural population and socioeconomic diversity, it offers a suitable region for studies on vaccine hesitancy and refusal. However, no case–control study conducted in Konya has examined the sociodemographic, behavioral, and vaccine confidence-related factors associated with childhood vaccine refusal.

This study aimed to identify sociodemographic, behavioral, and vaccine confidence-related factors associated with childhood vaccine refusal among parents with and without childhood vaccine refusal in Konya province.

## 2. Materials and Methods

### 2.1. Study Population and Sampling

This unmatched case–control study was conducted in accordance with the Strengthening the Reporting of Observational Studies in Epidemiology (STROBE) guidelines [10]. The data were collected from 1 July to 31 October 2025. The case (refusal) group consisted of parents registered at family health centers (the primary care units of the Turkish public health system) in Konya province with at least one child aged 0–4 years for whom they had refused at least one routine childhood vaccination, and the control (non-refusal) group included parents with children in the same age range who had completed all routine childhood vaccinations.

Vaccine refusal was operationally defined as parental refusal of at least one vaccine included in the Turkish National Immunization Program, which comprises Bacillus Calmette–Guérin (BCG), hepatitis B, diphtheria–tetanus–acellular pertussis-inactivated poliovirus–haemophilus influenzae type b (DTaP-IPV-Hib), pneumococcal conjugate vaccine (PCV), oral poliovirus vaccine (OPV), measles–mumps–rubella (MMR), varicella, and hepatitis A vaccines. The zero-to-four age group was preferred because this is when the vaccination schedule is most intensive, parental decision-making is active, and recall bias is minimized. To reduce selection bias, both the refusal and non-refusal groups were chosen from the same family health centers and similar geographical areas.

The source population consisted of parents of children aged 0–4 years registered at family health centers in Konya. Based on provincial health records, 10,073 children in this age group had at least one documented vaccine refusal and formed the sampling frame for the case group. Control participants were drawn from parents of children in the same age range registered at the same family health centers whose children had completed all routine vaccinations. The sample size was calculated using the OpenEpi program (version 3.01), targeting a minimum of 371 participants per group based on an assumed 50% unknown prevalence, 5% margin of error, and 95% confidence interval (CI) [11]. To account for non-response and incomplete questionnaires and to ensure sufficient statistical power for multivariable analyses, we aimed to recruit 421 participants from each group. Weighted invitation lists were prepared using a two-stage proportional approach based on provincial health records. First, participants were allocated according to district-level vaccine refusal frequencies; then, within each district, they were proportionally distributed across family health centers according to the number of registered refusal cases. For each selected family health center, the main participants were randomly selected, and a random backup list was created from the remaining eligible parents in the same center. Backup participants were invited when selected parents could not be reached, declined participation, or did not attend the family health center.

**Figure 1:**
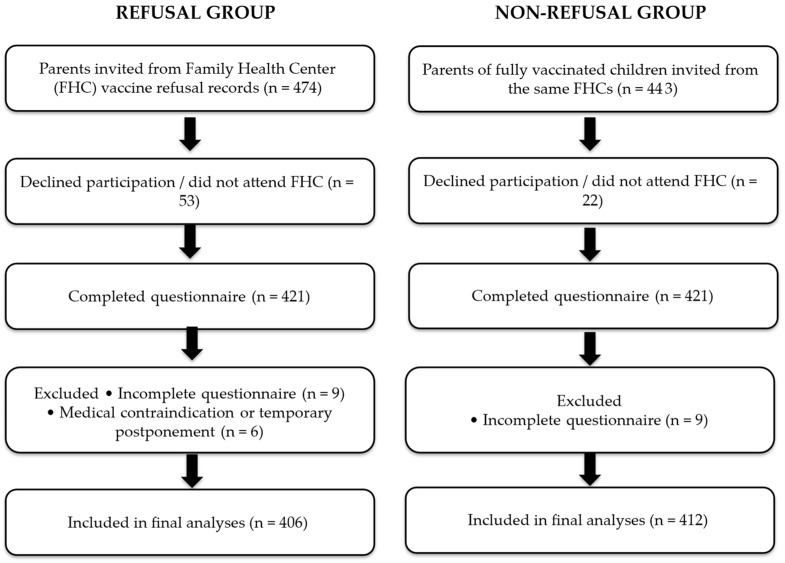
Flow diagram of the participant selection and inclusion processes.

Initially, 421 parents from each group were interviewed, and after excluding those with incomplete questionnaires and medical contraindications/deferrals, the final analyses were conducted with 406 refusal and 412 non-refusal participants (Figure 1). The participation rate was calculated as 91.8%.

### 2.2. Data Collection Tools and Procedure

A 47-item, mostly closed-ended data collection form was developed by the researchers. The form included questions on sociodemographic characteristics, vaccine knowledge, attitudes, behaviors, and preventive health services.

Vaccine refusal was defined based on family health center records. However, vaccine refusal is a behavior situated at the extreme end of vaccine hesitancy, and in the literature, vaccine hesitancy is considered a continuum [3,12]. Therefore, the 10-item Vaccine Hesitancy Scale developed by Larson et al. and adapted to Turkish by Aslan et al. was administered to both groups [3,13]. The scale is a 5-point Likert-type instrument, and items 5, 9, and 10 were reverse-coded during evaluation. The total score ranges from 10 to 50. Lower scores indicate lower vaccine confidence and higher vaccine hesitancy, whereas higher scores indicate higher vaccine confidence and lower vaccine hesitancy. Representative example items and response options from the Turkish validated version used in this study are provided in Supplementary Table S2. Permission to use the scale was obtained. The scale was used to compare the level of vaccine hesitancy between groups.

Data were collected through face-to-face interviews with parents at family health centers who were invited by phone. Participant confidentiality was maintained throughout the study. Questionnaires were coded using study identification numbers, and no personal identifiers were included in the analysis dataset. Completed forms were stored securely and were accessible only to the research team. Surveyors received standardized training, and a pilot study was conducted with 25 cases and 27 controls at one family health center. The pilot study participants were not included in the main analysis.

### 2.3. Statistical Analysis

The data were analyzed using IBM SPSS Statistics for Windows, version 22.0 (IBM Corp., Armonk, NY, USA). Normality of distribution for continuous variables was assessed using visual and analytical methods (Kolmogorov–Smirnov and Shapiro–Wilk tests). Continuous variables are presented as mean ± standard deviation and/or median (interquartile range), and categorical variables are presented as n (%). For comparisons between groups, the Mann–Whitney U test, Pearson chi-square test, and Fisher’s exact test were used as appropriate.

Multivariable logistic regression analysis was conducted to identify factors independently associated with childhood vaccine refusal. Candidate variables were selected based on univariate analysis (*p* < 0.20 as a screening threshold), prior literature, and conceptual relevance to vaccine refusal. Conceptual relevance referred to variables with a plausible theoretical or literature-based relationship with vaccine refusal, even if they did not meet the univariate screening threshold. For example, maternal education level was retained because of its frequently reported association with vaccine hesitancy in the literature.

Variables that directly measured attitudes very close to the outcome itself were not included in the multivariable model. For example, general trust in childhood vaccines and belief that childhood vaccines protect against diseases were excluded because they represent core components of vaccine refusal rather than independent background predictors. Including such variables in the model could make the interpretation of other associated factors less clear.

All selected variables were entered into the multivariable logistic regression model simultaneously. To avoid an overly complex model, the number of variables was limited before analysis according to the available number of vaccine refusal cases. We also examined whether the selected predictors were too highly correlated with each other by using variance inflation factors (VIFs). No problematic multicollinearity was observed, as all VIF values were below 2.0.

Results are presented as crude odds ratios (ORs), adjusted odds ratios (aORs), and 95% confidence intervals (CIs). The fit of the logistic regression model to the observed data was assessed using the Hosmer–Lemeshow goodness-of-fit test. Cox & Snell and Nagelkerke R^2^ values were reported to describe the overall explanatory performance of the model.

To test whether the main findings changed after removing the strongest perception-related variable, we repeated the multivariable logistic regression analysis without the variable “perceived vaccine-related adverse events in the social environment.” This analysis was performed because this variable may partly reflect vaccine-related beliefs shaped by previous attitudes or information exposure, rather than acting only as an independent background factor. Statistical significance was set at *p* < 0.05 for all analyses.

## 3. Results

A total of 818 parents participated in this study (406 cases, 412 controls). Of the participants, 90.1% (*n* = 737) were mothers, 8.6% (*n* = 70) were fathers, and 1.3% (*n* = 11) were grandmothers. In the refusal group (*n* = 406), 88.4% of respondents were mothers, 10.6% were fathers, and 1.0% were grandmothers, whereas in the non-refusal group (*n* = 412), these rates were 91.7%, 6.6%, and 1.7%, respectively. No significant difference was found between the study groups in terms of respondent type (*p* = 0.085).

### 3.1. Sociodemographic and Basic Characteristics of the Participants

The refusal and non-refusal groups were similar in terms of parental age, educational status, perceived income level, family type, residential region, smoking status, presence of chronic disease, and COVID-19 history (*p* > 0.05). However, the proportion of employed mothers was significantly higher in the non-refusal group (29.6% vs. 14.5%; *p* < 0.001). The number of children was significantly higher in the refusal group (2.2 ± 1.1 vs. 2.0 ± 1.0; *p* = 0.009) (Table 1).

In the non-refusal group, the rates of parents who received the COVID-19 vaccine (87.6% vs. 51.2%; *p* < 0.001), mothers who completed their own childhood vaccinations (84.7% vs. 72.7%; *p* < 0.001), and mothers who received the tetanus vaccine during pregnancy (90.5% vs. 66.5%; *p* < 0.001) were significantly higher compared to the refusal group (Table 1).

### 3.2. Vaccine Information Sources, Attitudes, and Preventive Behaviors

In the non-refusal group, the rates of obtaining information about childhood vaccinations from physicians (80.6% vs. 69.5%; *p* < 0.001) and other healthcare personnel were higher, whereas in the refusal group, the rates of obtaining information about childhood vaccinations from the internet/social media (52.5% vs. 19.9%; *p* < 0.001), written sources, television, friends/family, and religious leaders were significantly higher (Table 2).

Vitamin K administration to the child after birth was significantly higher in the non-refusal group (Table 2).

Trust in childhood vaccines (90.8% vs. 12.6%; *p* < 0.001) and the belief that vaccines protect against diseases were much higher in the non-refusal group. However, the proportion of those who reported observing serious side effects after vaccination in their surroundings was significantly higher in the refusal group (43.8% vs. 5.3%; *p* < 0.001). The proportion of those who believed that vaccination should be mandatory was 63.8% in the non-refusal group and only 3.2% in the refusal group (*p* < 0.001) (Table 2).

### 3.3. Vaccine Hesitancy Scale Results

**Figure 2:**
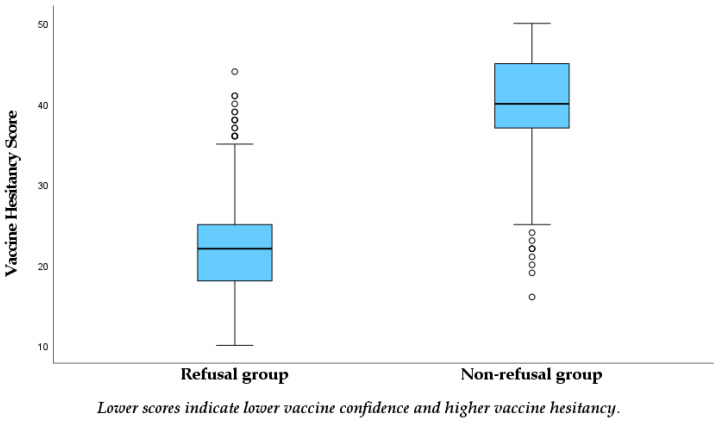
Distribution of Vaccine Hesitancy Scale scores according to childhood vaccine refusal status. Scores were significantly lower in the refusal group (Mann–Whitney U test, *p* < 0.001), indicating lower vaccine confidence and higher vaccine hesitancy. The boxes represent the interquartile range, the horizontal line indicates the median, whiskers represent values within 1.5 times the interquartile range, and dots indicate outliers.

Vaccine Hesitancy Scale scores were significantly lower in the refusal group than in the non-refusal group [22.2 ± 6.4/22 (18–25) vs. 39.8 ± 6.5/40 (36–45); *p* < 0.001] (Figure 2).

### 3.4. Multivariable Analysis Results

As shown in Table 3, multivariable logistic regression analysis demonstrated that maternal employment status (aOR = 0.371, *p* < 0.001), COVID-19 vaccination of either parent (aOR = 0.131, *p* < 0.001), complete childhood immunization history of the mother (aOR = 0.418, *p* < 0.001), tetanus vaccination during pregnancy (aOR = 0.259, *p* < 0.001), and postnatal vitamin K administration (aOR = 0.256, *p* < 0.001) were independently associated with lower odds of childhood vaccine refusal. In addition, the number of children was associated with higher odds of vaccine refusal (aOR = 1.281, *p* = 0.027). The strongest association was observed for parents who believed that a child in their surroundings had developed a serious adverse effect following vaccination (aOR = 16.982, *p* < 0.001).

### 3.5. Reasons for Vaccine Refusal and Decision Processes

**Figure 3:**
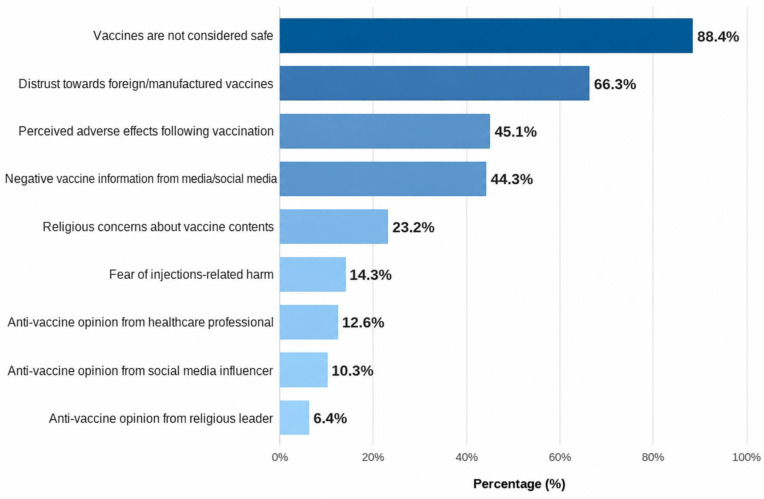
Distribution of reported reasons for childhood vaccine refusal among parents in the refusal group.

Characteristics of the vaccine refusal process are summarized in Table 4. As shown in Figure 3, the most frequently stated reason by parents who refused vaccination was the belief that vaccines are not safe (88.4%). This was followed by distrust toward vaccines produced in foreign countries (66.3%), perceived adverse effects following vaccination (45.1%), and negative vaccine information from social media (44.3%). The distribution of reported reasons for vaccine refusal among parents in the refusal group is presented in Figure 3. In addition, 79.6% of vaccination refusal decisions were reported to have been made jointly by both parents. Furthermore, 21.9% of parents in the refusal group stated that they had been advised by a healthcare professional not to vaccinate their children.

## 4. Discussion

This case–control study revealed that childhood vaccine refusal in Konya province was associated with maternal employment status, number of children, previous vaccination experiences, preventive health behaviors, sources of vaccine information, and vaccine confidence-related factors (Table 1, Table 2 and Table 3). These findings should not be interpreted as complete avoidance of preventive healthcare services among parents in the refusal group. Indeed, many parents in this group still reported acceptance of neonatal preventive practices, including vitamin K administration and newborn heel-prick screening. However, the uptake of these practices was significantly lower than in the non-refusal group, suggesting relatively reduced engagement with preventive healthcare services rather than absolute avoidance. This pattern highlights that vaccine hesitancy arises not only from a lack of information but also from multidimensional determinants, particularly vaccine confidence-related factors [3,12].

In this study, maternal employment was more frequent in the non-refusal group and was independently associated with lower odds of vaccine refusal (Table 1 and Table 3). Increased access to health services among working mothers, more opportunities for contact with healthcare professionals, stronger decision-making autonomy, and improved health literacy may partly explain this relationship. In contrast, a higher number of children was independently associated with increased odds of vaccine refusal (Table 1 and Table 3), possibly reflecting parental fatigue, time constraints, or cumulative negative experiences in larger families. In the sensitivity analysis, higher maternal education was associated with increased odds of childhood vaccine refusal (Supplementary Table S1), with a stepwise increase across education levels: mothers with high school education had 62% higher odds, and those with university or higher education had more than twice the odds of refusal compared with mothers with primary school education or less. This finding is consistent with the well-documented “education paradox” in vaccine hesitancy research, whereby more highly educated parents—despite often having greater health literacy in conventional terms—are more frequently exposed to alternative information sources, particularly internet-based and social media content, engage more actively in independent information-seeking, and may apply a critical-evaluation stance to official health recommendations [7,14,15,16]. As discussed in Section 4.1, this association was weaker in the main multivariable model after adjustment for perceived vaccine-related adverse events in the social environment, suggesting that risk perceptions may partly explain the relationship between maternal education and refusal.

This study also showed that vaccine refusal was associated with preventive practices during pregnancy and the neonatal period, including tetanus vaccination during pregnancy, postnatal vitamin K administration, and heel-prick blood screening. This finding suggests that vaccine refusal may be part of a broader pattern of reduced engagement with selected maternal and child preventive health services, rather than a single isolated decision. Consistent with the literature, the concept of “healthcare system engagement,” in which early acceptance of preventive services and subsequent childhood immunizations may reinforce each other, is also supported by this study [8,9,17,18].

Similarly, in the refusal group, greater reliance on the internet/social media and television as sources of information, together with lower use of physicians and other healthcare professionals as information sources, highlights the importance of vaccine information channels [5,6,19,20]. One of the most striking findings was the strong negative association between parental COVID-19 vaccination and childhood vaccine refusal. This suggests that vaccine hesitancy may extend across different vaccine types and age groups, and that mistrust emerging during the pandemic may also have adversely affected routine immunization programs [5,9,21,22]. Another noteworthy finding was that nearly one-fifth of parents in the refusal group reported being advised by a healthcare professional not to vaccinate their child. Although this finding may partly reflect subjective perceptions or communication misunderstandings, it remains important because healthcare professionals are among the most trusted and influential sources of vaccine-related information for the public. Previous studies have shown that healthcare professionals play a central role in shaping vaccine confidence and parental vaccination decisions, whereas vaccine hesitancy and concerns about vaccine safety exist among some healthcare professionals, particularly in the context of newly developed vaccines and their perceived adverse effects. Therefore, strengthening vaccine confidence and communication skills among healthcare professionals may play a critical role in reducing childhood vaccine refusal [23,24].

These findings have practical implications for vaccine communication strategies. Interventions should go beyond simply providing information and should focus on trust-building, transparent risk communication, and dialogue-based engagement by healthcare professionals. In addition, because non-professional information sources and social media were more frequently used by parents in the refusal group, proactive and evidence-based communication strategies on widely used media platforms may help address vaccine-related misinformation and risk perceptions.

Safety concerns and trust-related perceptions were prominent among parents in the refusal group. In this group, 88.4% of parents stated that vaccines are not safe, and 66.3% reported distrust toward foreign-manufactured vaccines. In addition, the perception of serious adverse effects after vaccination in the social environment was strongly associated with vaccine refusal in the multivariable model (aOR = 16.982), highlighting the important role of perceived vaccine risk in parental decision-making. This variable may also reflect pre-existing perceptions and beliefs about vaccines rather than objectively verified adverse events. These findings support the relevance of the “confidence” dimension in the WHO vaccine hesitancy model in the Konya sample [3,25,26,27,28].

### 4.1. Methodological Considerations and Sensitivity Analysis

Although the perception of vaccine-related adverse events in the social environment emerged as the strongest factor associated with vaccine refusal in the multivariable model (aOR = 16.982, 95% CI: 9.914–29.089), this finding warrants cautious interpretation. Because perception and refusal behavior were measured at the same time, it is not possible to determine whether perceived adverse events preceded vaccine refusal or whether parents who had already refused vaccination were more likely to recall or interpret events in their social environment as vaccine-related. Parents who have already refused vaccination may be more likely to recall or interpret illness episodes in their social environment as being related to prior vaccinations, particularly when they are exposed to information sources that reinforce such concerns. Therefore, this variable should be interpreted as a marker of perceived vaccine risk within a broader vaccine-related belief system rather than as an objectively verified exposure.

To examine whether the other independent associations remained stable, we repeated the multivariable model after excluding the strongest perception-related variable: perceived vaccine-related adverse events in the social environment. This additional analysis was also used to explore whether this perception variable might partly explain the association between background factors, such as maternal education, and vaccine refusal (Supplementary Table S1). The direction and statistical significance of associations for maternal employment, parental COVID-19 vaccination, maternal completion of childhood immunizations, tetanus vaccination during pregnancy, neonatal vitamin K administration, and number of children remained substantively unchanged, supporting the stability of these findings.

Importantly, however, maternal education level—non-significant in the main multivariable model—emerged as an independent risk factor for vaccine refusal in the sensitivity model, with a stepwise increase in the likelihood of refusal across higher education levels. This pattern suggests that perceived vaccine-related adverse events in the social environment may partly explain the association between maternal education and vaccine refusal. In the main multivariable model, this perception variable was included together with maternal education; after it was removed in the sensitivity analysis, the association between higher maternal education and vaccine refusal became stronger. This finding suggests that some of the association between maternal education and refusal may operate through education-related differences in information exposure and vaccine-related risk perceptions [29,30].

From a public health perspective, these perceptions are important because they may be modifiable. Transparent risk communication, correction of vaccine misinformation in community and online settings, and dialogue-based engagement by trusted healthcare professionals may help reduce the formation and reinforcement of perceived vaccine risks [25,27,28].

### 4.2. Strengths and Limitations of the Study

This study has several methodological strengths. First, it is among the limited number of investigations in Türkiye that have employed a case–control design to examine childhood vaccine refusal [5,6,7], allowing direct comparison of refusing and non-refusing parents from the same province and similar primary care settings. Second, as Konya is Türkiye’s largest province by area and encompasses both urban and rural populations with substantial socioeconomic heterogeneity, the findings provide robust regional-level insights with broader applicability. Third, the use of face-to-face interviews enabled an in-depth assessment of families’ knowledge, attitudes, behaviors, and information sources, while the high participation rate (91.8%) minimized non-response bias. Fourth, the additional sensitivity analysis showed that the main findings remained largely stable after removing the strongest perception-related variable. We also checked whether the variables included in the regression model provided overly overlapping information, and no such problem was detected.

In terms of limitations, several methodological considerations warrant attention. First, reliance on self-reported data may have introduced social desirability and recall biases, particularly for sensitive items concerning vaccine attitudes and healthcare-related behaviors. Second, voluntary participation may have excluded families with strong vaccine refusal attitudes who declined to participate, which may have led to a conservative estimate of the true magnitude of associations. Third, the fact that the study was conducted in a single province (Konya) limits its national generalizability, although the province’s socioeconomic and demographic heterogeneity partially mitigates this concern. Fourth, because attitudes, perceptions, and vaccine refusal were measured at the same time, the direction of some associations cannot be determined. For example, it is unclear whether perceived vaccine-related adverse events in the social environment contributed to vaccine refusal or whether parents who had already refused vaccination were more likely to recall or interpret such events as vaccine-related. Fifth, some variables included in the regression model may reflect a common pattern of engagement with preventive healthcare. For example, parental COVID-19 vaccination, maternal childhood immunization completion, tetanus vaccination during pregnancy, and newborn vitamin K administration may all represent broader acceptance of preventive health services. Therefore, the individual estimates for these variables should be interpreted within this broader context. Sixth, as 90.1% of respondents were mothers, with smaller proportions of fathers (8.6%) and grandmothers (1.3%), responses concerning maternal-specific variables may have been subject to minor information bias when reported by non-mother respondents. Although the case–control design is useful for identifying associations, longitudinal studies are needed to determine whether vaccine-related beliefs lead to refusal or whether refusal reinforces these beliefs over time.

## 5. Conclusions

This case–control study identified several sociodemographic, behavioral, preventive health-related, and vaccine confidence-related factors associated with childhood vaccine refusal among parents in Konya, Türkiye. Vaccine refusal was associated with lower maternal employment, a higher number of children, lack of parental COVID-19 vaccination, lower uptake of maternal and neonatal preventive health practices, greater reliance on non-professional information sources, and lower vaccine confidence. These findings suggest that interventions to reduce childhood vaccine refusal should focus not only on providing information but also on strengthening trust, improving healthcare-professional communication, and addressing vaccine-related risk perceptions. Future longitudinal studies are needed to confirm the direction of these associations and to evaluate targeted interventions.

## Figures and Tables

**Table 1:** Sociodemographic characteristics and general vaccination-related features of parents according to study groups.

Variables	Refusal (*n* = 406)	Non-Refusal (*n* = 412)	*p*-Value
Mother’s age (years)	32.1 ± 5.7/31.5 (28–36)	32.9 ± 6.3/32 (28–37)	0.127
Father’s age (years)	35.2 ± 6.6/35 (30–40)	35.5 ± 6.9/35 (30–40)	0.747
Mother’s employment status	59 (14.5)	122 (29.6)	<0.001 *
Father’s employment status	385 (94.8)	395 (95.9)	0.477
Mother’s education level			
Primary school or less	98 (24.1)	114 (27.7)	0.441
High school	134 (33.0)	123 (29.9)	
University or higher	174 (42.9)	175 (42.5)	
Father’s education level			
Primary school or less	107 (26.4)	101 (24.5)	0.323
High school	114 (28.1)	102 (24.8)	
University or higher	185 (45.6)	209 (50.7)	
Perceived household income level			
Income less than expenses	130 (32.0)	153 (37.1)	0.190
Income equal to expenses	220 (54.2)	215 (52.2)	
Income greater than expenses	56 (13.8)	44 (10.7)	
Number of children	2.2 ± 1.1/2 (1–3)	2.0 ± 1.0/2 (1–3)	0.009 *
Family type			
Nuclear family	381 (93.8)	379 (92.0)	0.302
Extended family	25 (6.2)	33 (8.0)	
Place of residence			
Urban	400 (98.5)	400 (97.1)	0.162
Rural	6 (1.5)	12 (2.9)	
Smoking status of any parent	190 (46.8)	189 (45.9)	0.791
Chronic disease in any parent	84 (20.7)	75 (18.2)	0.369
History of COVID-19 in any parent	225 (55.4)	256 (62.1)	0.051
COVID-19 vaccination status of any parent	208 (51.2)	361 (87.6)	<0.001 *
Loss of a relative due to COVID-19	111 (27.3)	103 (25.0)	0.446
Mother’s childhood vaccinations complete	295 (72.7)	349 (84.7)	<0.001 *
Father’s childhood vaccinations complete	284 (70.0)	304 (73.8)	0.222
Mother received tetanus vaccine during pregnancy	270 (66.5)	373 (90.5)	<0.001 *

Categorical variables are presented as *n* (column percentage), and continuous variables are presented as mean ± standard deviation/median (interquartile range). * Statistically significant values.

**Table 2:** Distribution of parents’ vaccination information sources, attitudes, and preventive health behaviors by study groups.

Variables	Refusal (*n* = 406)	Non-Refusal (*n* = 412)	*p*-Value
Sources of information on childhood vaccinations			
Physician	282 (69.5)	332 (80.6)	<0.001 *
Other healthcare personnel	260 (64.0)	342 (83.0)	<0.001 *
Written sources (books, journals, newspapers, etc.)	152 (37.4)	71 (17.2)	<0.001 *
Internet/Social media	213 (52.5)	82 (19.9)	<0.001 *
Television	38 (9.4)	17 (4.1)	0.003 *
Friends/Family recommendation	99 (24.4)	37 (9.0)	<0.001 *
Religious leaders	15 (3.7)	2 (0.5)	0.001 *
General trust in childhood vaccinations	51 (12.6)	374 (90.8)	<0.001 *
Belief that childhood vaccinations protect against diseases	82 (20.2)	379 (92.0)	<0.001 *
Having a serious illness due to not being vaccinated in the surroundings	18 (4.4)	51 (12.4)	<0.001 *
Belief that there is a child who developed a serious side effect after vaccination in the surroundings	178 (43.8)	22 (5.3)	<0.001 *
Belief that vaccination should be mandatory			
Agrees	13 (3.2)	263 (63.8)	<0.001 *
Does not agree	344 (84.7)	102 (24.8)	
No opinion	49 (12.1)	47 (11.4)	
Newborn received vitamin K after birth	334 (82.3)	394 (95.6)	<0.001 *
Having newborn heel-prick screening (NHS) performed	371 (91.4)	408 (99.0)	<0.001 *
Belief that they would have their child vaccinated against a serious and fatal disease if not previously vaccinated	108 (26.6)	324 (78.6)	<0.001 *

Data are presented as n (column percentage). * Statistically significant values.

**Table 3:** Multivariable logistic regression analysis of independent factors associated with childhood vaccine refusal.

Variables	OR	95% CI	*p*-Value	aOR	95% CI	*p*-Value
Mother’s age (years)	0.979	0.956–1.001	0.063	0.983	0.946–1.022	0.394
Mother’s education level						
Primary school or less		Reference			Reference	
High school	1.267	0.880–1.824	0.203	1.317	0.797–2.177	0.282
University or higher	1.157	0.822–1.628	0.404	1.682	0.985–2.871	0.057
Mother’s employment status	0.404	0.285–0.572	<0.001 *	0.371	0.218–0.633	<0.001 *
Perceived household income level						
Income less than expenses		Reference			Reference	
Income equal to expenses	1.204	0.892–1.626	0.225	1.319	0.877–1.985	0.184
Income greater than expenses	1.498	0.947–2.370	0.084	1.236	0.657–2.325	0.511
Number of children	1.214	1.060–1.389	0.005 *	1.281	1.028–1.596	0.027 *
Any parent received COVID-19 vaccine	0.148	0.104–0.211	<0.001 *	0.131	0.086–0.200	<0.001 *
Mother’s childhood vaccinations complete	0.480	0.339–0.678	<0.001 *	0.418	0.262–0.667	<0.001 *
Mother received tetanus vaccine during pregnancy	0.208	0.141–0.306	<0.001 *	0.259	0.159–0.421	<0.001 *
Newborn received vitamin K after birth	0.212	0.124–0.362	<0.001 *	0.256	0.132–0.497	<0.001 *
Belief that there is a child who developed a serious side effect after vaccination in the surroundings	13.840	8.632–22.190	<0.001 *	16.982	9.914–29.089	<0.001 *

aOR: adjusted odds ratio; CI: confidence interval. Binary logistic regression was used. Dependent variable: childhood vaccine refusal. Hosmer–Lemeshow *p* = 0.904; Cox & Snell R^2^ = 0.399; Nagelkerke R^2^ = 0.502. * Statistically significant values.

**Table 4:** Characteristics of the vaccine refusal process among parents in the refusal group.

(*n* = 406)	*n* (%)
Person who decided not to vaccinate the child	
Both parents together	323 (79.6)
Mother	58 (14.3)
Father	16 (3.9)
Other *	9 (2.2)
Receiving advice from a healthcare professional not to vaccinate the child	89 (21.9)
Healthcare professional who provided the advice	
Family health center midwife/nurse	70 (17.2)
Family physician	38 (9.4)
Pediatrician	13 (3.2)
Other	10 (2.5)
Would have the child vaccinated against rabies in case of cat/dog bite	334 (82.3)
Would have the child vaccinated against tetanus in case of any injury	289 (71.2)
Positive attitude toward being invited by a healthcare professional for vaccination information	97 (23.9)
Would change their mind if faced with any sanction for not vaccinating the child	45 (11.1)

Data are presented as *n* (%). * Other: grandmother, grandfather, aunt, etc.

## Data Availability

The de-identified datasets generated and analyzed during this study are available from the corresponding author upon reasonable request, subject to ethical and institutional restrictions on participant confidentiality.

## Supplementary Materials

The following supporting information can be downloaded at: https://www.mdpi.com/article/10.3390/vaccines14060538/s1, File S1: STROBE checklist for case–control studies; Table S1: Sensitivity analysis excluding perceived vaccine-related adverse events in the social environment; Table S2: Representative example items and response options from the Vaccine Hesitancy Scale.
