# Supplementary material for: Childhood Vaccine Refusal: Sociodemographic, Behavioral, and Vaccine Confidence Factors in Konya, Türkiye
Source: Vaccines (Basel). 2026 Jun 17;14(6):538. doi: 10.3390/vaccines14060538 (PMC13307600; doi:10.3390/vaccines14060538)
Supplement: Supplementary file 1 [file vaccines-14-00538-s001.zip › vaccines-4356025-Supplementary_Table_S2.pdf]

Supplementary Table S2. Representative Example Items and Response Options from the Vaccine Hesitancy Scale

Representative example items from the validated Turkish version of the Vaccine Hesitancy Scale used in this study are shown below. The full validated scale was not reproduced; the table is intended to illustrate the item structure and the 5-point Likert response format.

No.	Representative example item	Response options
1	Childhood vaccines are important for my child’s health.	1 = Strongly disagree; 2 = Disagree; 3 = Neither agree nor disagree; 4 = Agree; 5 = Strongly agree
2	Childhood vaccines are effective.	Same 5-point Likert response options
5	New vaccines carry more risks than older vaccines.	Same 5-point Likert response options
9	I am concerned about serious adverse effects of vaccines.	Same 5-point Likert response options

Note. The scale was administered as a 5-point Likert-type instrument. Items 5, 9, and 10 were reverse coded during scoring. Higher total scores indicate greater vaccine confidence and lower vaccine hesitancy, whereas lower scores indicate lower vaccine confidence and higher vaccine hesitancy.
