# Supplementary material for: Childhood Vaccine Refusal: Sociodemographic, Behavioral, and Vaccine Confidence Factors in Konya, Türkiye
Source: Vaccines (Basel). 2026 Jun 17;14(6):538. doi: 10.3390/vaccines14060538 (PMC13307600; doi:10.3390/vaccines14060538)
Supplement: Supplementary file 1 [file vaccines-14-00538-s001.zip › vaccines-4356025-Supplementary_File_S1_STROBE_Checklist.pdf]

STROBE Checklist for Case-Control Studies

Title of Manuscript: Childhood Vaccine Refusal: Sociodemographic, Behavioral, and Vaccine Confidence Factors

Section	Item	Recommendation	Reported on Page
**Title and Abstract**	1a	Indicate the study design with a commonly used term in the title or abstract	Title page, Abstract
**Title and Abstract**	1b	Provide an informative and balanced summary of what was done and found	Abstract
**Introduction**	2	Explain the scientific background and rationale	Introduction, p. 1–2
**Introduction**	3	State specific objectives and hypotheses	Introduction, p. 2
**Methods**	4	Present key elements of study design early in the paper	Methods, p. 3
**Methods**	5	Describe setting, locations, and relevant dates	Methods, p. 3
**Methods**	6a	Give eligibility criteria, sources and methods of case ascertainment and control selection	Methods, pp. 3–4
**Methods**	6b	Give rationale for the choice of cases and controls	Methods, p. 3
**Methods**	7	Clearly define all outcomes, exposures, predictors, confounders	Methods, pp. 3–5
**Methods**	8	Give sources of data and details of assessment methods	Methods, p. 4
**Methods**	9	Describe efforts to address potential sources of bias	Methods, pp. 3–5 and Limitations
**Methods**	10	Explain how study size was determined	Methods, p. 3
**Methods**	11	Explain handling of quantitative variables in analyses	Methods, p. 5
**Methods**	12a	Describe all statistical methods, including confounding control	Methods, p. 5
**Methods**	12b	Describe methods used to examine subgroups/interactions	Not applicable
**Methods**	12c	Explain how missing data were addressed	Methods, p. 4 and Figure 1
**Methods**	12d	Explain matching criteria if applicable	Not applicable
**Methods**	12e	Describe sensitivity analyses	Not performed
**Results**	13a	Report numbers at each stage of study	Figure 1
**Results**	13b	Give reasons for non-participation	Figure 1
**Results**	13c	Consider use of a flow diagram	Figure 1
**Results**	14a	Give characteristics of study participants	Results, pp. 6–7 and Table 1

**Results**	14b	Indicate missing data for each variable	Figure 1
**Results**	15	Report numbers in each exposure category	Tables 1–4
**Results**	16a	Give unadjusted and adjusted estimates with precision	Table 3
**Results**	16b	Report category boundaries when continuous variables categorized	Tables 1 and 3
**Results**	16c	Translate relative risk estimates if relevant	Not applicable
**Results**	17	Report other analyses performed	Not applicable
**Discussion**	18	Summarize key results with reference to objectives	Discussion, p. 9
**Discussion**	19	Discuss limitations and potential bias	Discussion, pp. 10–11
**Discussion**	20	Give cautious interpretation considering limitations	Discussion, pp. 9–11
**Discussion**	21	Discuss generalizability	Discussion, p. 11
**Other Information**	22	Give source of funding and role of funders	Funding

Prepared according to the STROBE Statement checklist for case-control studies.
