# Supplementary material for: Childhood Vaccine Refusal: Sociodemographic, Behavioral, and Vaccine Confidence Factors in Konya, Türkiye
Source: Vaccines (Basel). 2026 Jun 17;14(6):538. doi: 10.3390/vaccines14060538 (PMC13307600; doi:10.3390/vaccines14060538)
Supplement: Supplementary file 1 [file vaccines-14-00538-s001.zip › vaccines-4356025-Supplementary_Table_S1.pdf]

Supplementary Table S1. Multivariable Logistic Regression Analysis of Independent Factors Associated with Childhood Vaccine Refusal

Variables	OR	95% CI	p-value	aOR	95% CI	p-value
**Mother's age (years)**	0.979	0.956–1.001	0.063	0.970	0.937-1.004	0.086
**Mother's education level**						
Primary school or less		*Reference*			*Reference*	
High school	1.267	0.880–1.824	0.203	1.621	1.028–2.555	0.038*
University or higher	1.157	0.822–1.628	0.404	2.381	1.470–3.858	<0.001*
**Mother's employment status**	0.404	0.285–0.572	<0.001*	0.364	0.227–0.585	<0.001*
**Perceived household income level**						
Income less than expenses		*Reference*			*Reference*	
Income equal to expenses	1.204	0.892–1.626	0.225	1.240	0.861–1.787	0.248
Income greater than expenses	1.498	0.947–2.370	0.084	1.639	0.931–2.885	0.087
**Number of children**	1.214	1.060–1.389	0.005*	1.393	1.143–1.699	0.001*
**Any parent received COVID-19 vaccine**	0.148	0.104–0.211	<0.001*	0.150	0.102–0.220	<0.001*
**Mother's childhood vaccinations complete**	0.480	0.339–0.678	<0.001*	0.453	0.297–0.690	<0.001*
**Mother received tetanus vaccine during pregnancy**	0.208	0.141–0.306	<0.001*	0.271	0.175–0.419	<0.001*
**Receiving vitamin K after birth**	0.212	0.124–0.362	<0.001*	0.296	0.160–0.545	<0.001*

aOR: adjusted odds ratio; CI: confidence interval. Binary logistic regression was used. Dependent variable: childhood vaccine refusal; Hosmer-Lemeshow p=0.509, Cox & Snell R^2: 0.278; Nagelkerke R^2: 0.371; *p<0.05 *Sensitivity analysis: identical to the primary multivariable model (Table 3), excluding the variable 'Belief that there is a child who developed a serious side effect after vaccination in the surroundings' to assess robustness of independent associations and explore potential over-adjustment effects.*
